# Congruence between co-occurrence and trait-based networks is scale-dependent: a case study with flea parasites of small mammalian hosts

**DOI:** 10.1017/S0031182024000969

**Published:** 2024-07

**Authors:** Boris R. Krasnov, Irina S. Khokhlova, Natalya P. Korallo-Vinarskaya, Anne Laudisoit, M. Fernanda López Berrizbeitia, Sonja Matthee, Julliana P. Sanchez, Michal Stanko, Luther van der Mesht, Maxim V. Vinarski

**Affiliations:** 1 Mitrani Department of Desert Ecology, Swiss Institute for Dryland Environmental and Energy Research, Jacob Blaustein Institutes for Desert Research, Ben-Gurion University of the Negev, Sede Boqer Campus, Midreshet Ben-Gurion, Israel; 2 French Associates Institute for Agriculture and Biotechnology of Drylands, Jacob Blaustein Institutes for Desert Research, Ben-Gurion University of the Negev, Sede Boqer Campus, Midreshet Ben-Gurion, Israel; 3 Laboratory for the Study of Parasitic Arthropods, Zoological Institute of Russian Academy of Sciences, Saint-Petersburg, Russian Federation; 4 EcoHealth Alliance, New York, NY 10018, USA; 5 Peveco GROUP, University of Antwerp, Belgium; 6 Programa de Conservación de los Murciélagos de Argentina (PCMA) and Instituto de Investigaciones de Biodiversidad Argentina (PIDBA)-CCT CONICET Noa Sur (Consejo Nacional de Investigaciones Científicas y Técnicas), Facultad de Ciencias Naturales e IML, UNT, and Fundación Miguel Lillo, San Miguel de Tucumán, Argentina; 7 Department of Conservation Ecology and Entomology, Stellenbosch University, Matieland, South Africa; 8 Centro de Investigaciones y Transferencia del Noroeste de la Provincia de Buenos Aires-CITNOBA (UNNOBA-UNSAdA- CONICET), Pergamino, Argentina; 9 Institute of Parasitology and Institute of Zoology, Slovak Academy of Sciences, Kosice, Slovakia; 10 Department of Zoology and Entomology, University of the Free State, Bloemfontein, South Africa; 11 Laboratory of Macroecology and Biogeography of Invertebrates, Saint-Petersburg State University, Saint-Petersburg, Russian Federation

**Keywords:** biogeographic realms, co-occurrence, fleas, mammals, networks, traits

## Abstract

We applied a novel framework based on network theory and a concept of modularity that estimates congruence between trait-based ( = functional) co-occurrence networks, thus allowing the inference of co-occurrence patterns and the determination of the predominant mechanism of community assembly. The aim was to investigate the relationships between species co-occurrence and trait similarity in flea communities at various scales (compound communities: across regions within a biogeographic realm or across sampling sites within a geographic region; component communities: across sampling sites within a geographic region; and infracommunities: within a sampling site). We found that compound communities within biogeographic realms were assembled *via* environmental or host-associated filtering. In contrast, functional and spatial/host-associated co-occurrence networks, at the scale of regional compound communities, mostly indicated either stochastic processes or the lack of dominance of any deterministic process. Analyses of congruence between functional and either spatial (for component communities) or host-associated (for infracommunities) co-occurrence networks demonstrated that assembly rules in these communities varied among host species. In component communities, stochastic processes prevailed, whereas environmental filtering was indicated in 4 and limiting similarity/competition in 9 of 31 communities. Limiting similarity/competition processes dominated in infracommunities, followed by stochastic mechanisms. We conclude that assembly processes in parasite communities are scale-dependent, with different mechanisms acting at different scales.

## Graphical abstract

**Figure:**
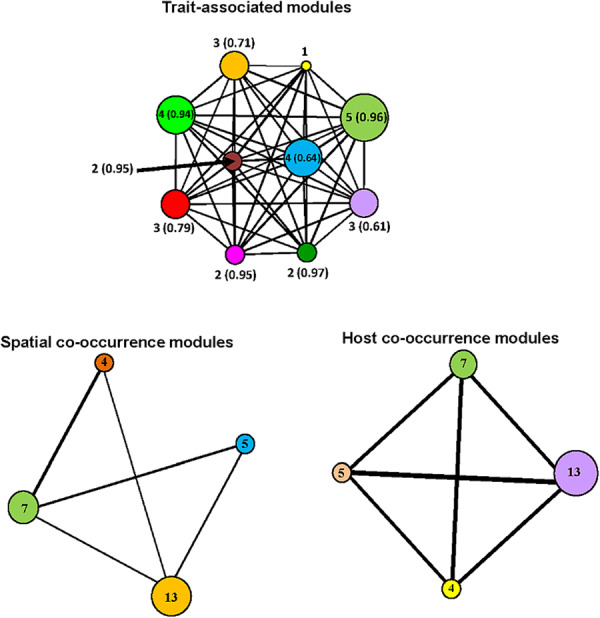

## Introduction

Species associations within a metacommunity (i.e. a set of biological communities occupying multiple localities and potentially linked by dispersal; Wilson, 1992; Leibold and Mikkelson, 2002) may be either random or non-random (e.g. Diamond, 1975; Connor and Simberloff, 1979; Gotelli, 2000; Gotelli and McCabe, 2002; Ulrich, 2004; Gotelli and Ulrich, 2010). Non-random species associations indicate that communities within a metacommunity are structured, that is, variation in species composition across communities is, to a certain degree, predictable, and communities are thus organized by certain assembly rules (e.g. Diamond, 1975; Patterson and Atmar, 1986). The non-randomness of species associations (=co-occurrence pattern) is tested using null models (Gotelli, 2000). In particular, if the frequency of co-occurrence is greater than expected by chance, then these species are positively associated, whereas if this frequency is lower than expected by chance, then these species are negatively associated (Gotelli, 2000; Gotelli and McCabe, 2002). Alternatively, if the frequency of co-occurrence does not differ from that expected by chance, then the community is randomly assembled.

The pattern of species co-occurrence allows inferring the mechanisms of community structuring. For example, predominantly positive co-occurrences suggest a mechanism resembling environmental filtering in which abiotic and/or biotic factors prevent the establishment or persistence of species in a particular location (Maire *et al*. 2012; Kraft *et al*. 2015). As a result, an environmental filter allows a community to contain only species possessing certain traits that are necessary for persistence in that environment (e.g. Ingram and Shurin, 2009). Consequently, co-occurring species are expected to be similar in their traits. Predominantly negative co-occurrences may indicate interspecific competition (Diamond, 1975) or the ‘ghost of competition past’, both resulting in the dissimilarity of co-occurring species in their traits (limiting similarity; MacArthur and Levins, 1967). The latter concept states that species can coexist only if their overlap in resource use is limited due to morphological, ecological or behavioural dissimilarities. However, Ulrich and Gotelli (2013) argued that negative co-occurrences may result from a change in species composition (species turnover) determined by limited dispersal and/or a gradient in habitat quality, independently of trait similarity or dissimilarity. Nevertheless, the majority of the above-cited studies suggest that studies aimed at inferring the community assembly rules from the pattern of species co-occurrences should incorporate information on species traits.

Although patterns of species co-occurrence have been studied in a variety of biological communities (e.g. Gotelli and McCabe, 2002; Jenkins, 2006; Ulrich and Zalewski, 2006; Veech, 2006; Ulrich and Gotelli, 2007; Ulrich *et al*. 2017; Freilich *et al*. 2018; Harikrishnan and Vasudevan, 2018), species trait information has rarely been incorporated in these studies (Korňan *et al*. 2019; Legras *et al*. 2019; Vinarski *et al*. 2020). Furthermore, the absolute majority of species co-occurrence studies considered pairwise co-occurrences. However, not every pair of species in a community is positively or negatively associated, but rather, a general non-random pattern arises due to associations of a subset of species only (Sfenthourakis *et al*. 2006; Veech, 2006; Gotelli and Ulrich, 2010). Therefore, consideration of groupwise, rather than pairwise, co-occurrences will likely improve pattern detection and will make inferring assembly rules more reliable (Morueta-Holme *et al*. 2016; Legras *et al*. 2019). This can be achieved by applying a network approach to species co-occurrence studies. Recently, Legras *et al*. (2019) proposed a new methodological approach based on the concepts of network theory and modularity and aimed to disentangle the deterministic and stochastics drivers of species co-occurrence patterns accounting for multiple functional traits. In brief, this approach involves calculating modularity in (a) a site × species network and (b) a trait × species network and then assessing the congruence between the 2 networks by testing whether the species from a given functional module belong to the same co-occurrence module. Legras *et al*. (2019) demonstrated that this approach can accurately detect assembly rules acting at different spatial scales.

The scale-dependence (spatial or temporal) of co-occurrence patterns has been found in multiple taxa (Gotelli and Ellison, 2002; Sanders *et al*. 2007; Harms and Dinsmore, 2016; Legras *et al*. 2019). However, one of the methodological difficulties in community ecology studies is to identify community boundaries, which often cannot be unequivocally defined (Strayer *et al*. 2003). This is especially true for communities at different scales. From this perspective, parasite communities present convenient models for elucidating the patterns of species co-occurrence and underlying processes. This is because parasite community boundaries, both in space and time, can be identified more easily than those of free-living species, due to the fact that parasites inhabit a set of ‘islands’ represented by their hosts, whereas the environment between these ‘islands’ is mostly unfavourable. Furthermore, parasite communities are fragmented between host individuals (=infracommunities), populations of conspecific hosts (=component communities), and communities of multiple host species (=compound communities) (Holmes and Price, 1986; Poulin, 2007).

A pioneering study of species co-occurrence in parasite communities dealt with infracommunities of ectoparasites in marine fishes and reported an absence of any strong pattern, thus suggesting that these communities are mostly randomly assembled (Gotelli and Rohde, 2002), although the opposite appeared to be the case for ectoparasites of freshwater fishes (Bellay *et al*. 2012). Studies of co-occurrence patterns in ectoparasitic arthropods of terrestrial hosts reported predominantly positive species associations in different taxa, host species, and geographic regions (Krasnov *et al*. 2006*a*, 2010, 2011, 2019; Presley, 2007; Tello *et al*. 2008; Veitch *et al*. 2020). By comparison, to the best of our knowledge, only one study has incorporated information on parasite traits in investigating their co-occurrence patterns (Vinarski *et al*. 2020). This study compared the probability of pairwise co-occurrences and pairwise trait dissimilarity in communities of fleas and gamasid mites, parasitic on small mammals. A significant, albeit weak, tendency of flea, but not mite, communities to be composed of functionally similar species has been found. However, using pairwise co-occurrences and trait similarity could bias the results due to the above-mentioned reason that a detection of non-randomness could be associated with only a subset of the entire community species pool (e.g. Sfenthourakis *et al*. 2006).

Here, we aimed to remedy this shortcoming and applied the framework proposed by Legras *et al*. (2019) (see above) to investigate the relationships between species co-occurrence and trait similarity in flea communities at various scales (compound communities: across regions within a biogeographic realm or across sampling sites within a geographic region; component communities: across sampling sites within a geographic region; and infracommunities: within a sampling site). Fleas are holometabolous haematophagous insects, being the most diverse on small and medium-sized burrowing mammals. In the absolute majority of species, larvae are not parasitic and feed on all kinds of organic matter found in the hosts’ burrows/nests where pre-imaginal development takes place (Marshall, 1981; Krasnov, 2008). Based on the predominantly aggregative structure of flea communities found in earlier studies (e.g. Krasnov *et al*. 2006*a*, 2011) and the link between pairwise co-occurrence and trait similarity, we expected to find congruence between modules of co-occurrence and functional networks (see below definition of modules) at all hierarchical scales. For compound communities, we considered flea species co-occurrences not only among regions or sites but also among host species. In addition, we inferred the main processes (deterministic ones, such as limiting similarity/competition and environmental filtering, or stochastic ones) acting on community assembly.

## Materials and methods

### Data on flea species distribution

Data on flea species distribution across regions and host species within biogeographic realms (the Afrotropics, Australasia, Indo-Malay, Nearctic, Neotropics, and Palearctic) were obtained from various literature sources. At this scale (i.e. within a biogeographic realm), only host species that harboured fleas and belonged to tachyglossid Monotremata, Dasyuromorphia, Paramelemorphia, Diprotodontia, Macropodiformes, Didelphimorphia, Paucituberculata, Macroscelidea, Eulipotyphla, Rodentia and the ochotonid Lagomorpha were considered. This study included data on the distribution of 1313 flea species across 1153 mammal host species in 15 Afrotropical regions, 8 Australasian regions, 10 Indo-Malayan regions, 23 Nearctic regions, 17 Neotropical regions and 36 Palearctic regions (see lists of regions, maps and references in Krasnov *et al*. 2022 and lists of flea and host species in the respective publications).

At the scale of regional compound communities, component communities and infracommunities, the sources of data were surveys of fleas on individual small mammals carried out in 1981–1984 across 83 sampling sites in Mongolia (Kiefer *et al*. 1982, 1984, 1990); in 1998–2015 across 38 sampling sites in Northwest Argentina (López Berrizbeitia, 2018; López Berrizbeitia and Díaz, 2019); in 2006–2013 across 22 sites in Argentinian Patagonia (Sanchez, 2012; Sanchez and Lareschi, 2013, 2019); in 1986–2000 across 13 sites in eastern Slovakia (Stanko *et al*. 2002); in 1988 across 49 sites in Western Siberia [compiled from the database of the Omsk Research Institute of Natural Foci Infections (Omsk, Russian Federation) by NPK-V] (Surkova *et al*. 2018); in 2003–2018, across 36 sites in southwestern South Africa (Matthee *et al*. 2007; van der Mescht and Matthee, 2017; Stevens *et al*. 2022); and in 2005–2008 across 18 sites in the Lushoto District, Usambara Mountains, Tanzania (Laudisoit *et al*. 2009*a*, 2009*b*). Lists of flea and mammal species in each region can be found in Supplementary Material, Appendix 1, Table S1.

At the scale of component communities, 31 host species harbouring at least 8 flea species were selected from the regional data (see Supplementary Material, Appendix 1, Table S2). At the scale of infracommunities, 25 host species harbouring at least 6 flea species at least 1 sampling site were selected from the regional data (see Supplementary Material, Appendix 1, Table S3). The latter analyses were carried out for conspecific host individuals captured at the same sampling site at the same time.

### Data on flea traits

Although a trait, by definition, is ‘any morphological, physiological or phenological feature measurable at the individual level’ (Violle *et al*. 2007), information on parasite traits, measurable at the individual level, is largely unavailable. Consequently, we used species-specific attributes or characteristics that we further refer to as traits for simplicity. As such, species-specific attributes have been used in earlier studies of functional diversity (e.g. feeding habit in Pavoine *et al*. 2016). In the current study, each flea species was characterized by 4 ecological and 2 morphological traits. Ecological traits included the total number of host species exploited across a flea's geographic range; these hosts’ phylogenetic diversity; the latitudinal span of geographic range; and microhabitat preference as the relative time spent either in the hair of the host or in its/their nest/burrow (preference for hair or for nest, or else no clear preference). Morphological traits included the number and possession of sclerotized ctenidia and body size. The rationale for selecting these traits, information sources and calculation details can be found elsewhere (Krasnov *et al*. 2015, 2024). Data on the latitudinal span of geographic range were not available for the Australasian and the Indo-Malayan fleas, and data on microhabitat preference were not available for the Australasian fleas.

### Data analyses

As mentioned above, the main idea of Legras *et al*. (2019) is to compare the functional and the co-occurrence networks when the former is based on species traits and the latter is based on the number of species co-occurrences. In this case, the edges within each network represent similarity in either species traits or species co-occurrences, respectively. In a modularly structured network, species from some distinct groups ( = modules) are connected mainly with each other, while they have much fewer connections to other species belonging to other modules. Consequently, modularity measures the density of edges inside groups compared to edges between groups (Newman, 2006). In the framework of Legras *et al*. (2019), each network's modularity is calculated, and the modules’ species compositions are compared between the 2 networks, that is, testing whether species from a given functional module also belong to a co-occurrence module. If this is the case, then functionally similar species inhabit/are found in the same sites/regions/host species. This would strongly suggest that environmental or host-associated factors act as filters determining species composition. On the contrary, high dissimilarity between the 2 networks would suggest that functionally similar species are distributed among different sites/regions/host species, likely resulting from interspecific competition that, in turn, leads to reduced similarity between co-occurring species (i.e. limiting similarity *sensu* MacArthur and Levins, 1967). However, a congruence between the module species composition of the functional and the co-occurrence networks that is not especially high and not especially low would indicate that species distribution among sites/regions/host species does not differ from random. This situation results either from stochastic mechanisms (e.g. dispersal limitations and/or population dynamics; Hubbell, 2001) or from the fact that deterministic processes (environmental filtering and competition) act simultaneously, affecting species co-occurrences in the opposite directions. The latter may occur if the distribution of some species in a metacommunity is caused by environmental/host-associated filters, whereas the distribution of other species is determined by competition. In such a case, there is no clear dominance of one of the processes, so they cannot be disentangled.

In our study, the approach of Legras *et al*. (2019) was slightly modified. Although the construction of co-occurrence networks and identification of co-occurrence modules followed Legras *et al*. (2019), the construction of functional networks and identification of functional modules (=functional groups) was carried out using the best partitioning of the functional trait space and followed Rubio and Swenson (2022).

A functional matrix (flea species × traits with traits in columns) was compiled for all 1362 fleas in the dataset. Two presence/absence co-occurrence matrices for compound communities (either realm-specific or regional) and 1 presence/absence co-occurrence matrix for either component communities or infracommunities were constructed (see scheme in Supplementary Material, Appendix 2). For each compound community at the scale of a biogeographic realm (i.e. within a realm), these matrices were (a) a spatial co-occurrence matrix: flea species × regions (regions in columns) and (b) a host co-occurrence matrix: flea species × host species (host species in columns). For each compound community at the regional scale (i.e. within a region), these matrices were (a) a spatial co-occurrence matrix: flea species × sampling sites (sites in columns) and (b) a host co-occurrence matrix: flea species × host species (host species in columns). For each set of component communities (i.e. within a host species within a region across sampling sites), a spatial co-occurrence matrix was flea species × sampling sites (sites in columns). For each set of infracommunities (e.g. within a host species at the same sampling site across host individuals), a host co-occurrence matrix was flea species × host individuals. Although Legras *et al*. (2019) recommended constructing the co-occurrence matrices using species abundances, presence/absence data appeared to be more reliable than abundance data for parasites (Gotelli and Rohde, 2002). This is because (a) parasites are aggregated across host individuals, being the most abundant only in a few host individuals, whereas the majority of host individuals harbour only a few or no parasites (Anderson and May, 1978); (c) ectoparasite counts are an unreliable measure of their abundance (see Krasnov *et al*. 2006*b*), and (b) comparison of the results of analyses using abundance *vs* presence/absence data in studies of parasite community ecology, demonstrated similar performances of these analyses (Haukisalmi and Henttonen, 1993; Krasnov *et al*. 2021; but see Brian and Aldridge, 2021).

Then, a similarity matrix was calculated for each above-mentioned matrix, that is, a matrix of trait (=functional) similarity between species from a functional matrix and a matrix(ces) of co-occurrence similarity from co-occurrence matrix(ces). Functional similarity was calculated as (1-Gower distance index) for each species pair, using the ‘FD’ package (Laliberté, and Legendre, 2010; Laliberté *et al*. 2014), implemented in the R statistical environment (R Core Team, 2023). Co-occurrence similarity was calculated as (1-binary version of the Bray–Curtis dissimilarity index), using the R package ‘vegan’ (Oksanen *et al*. 2022). The values of these similarity matrices were then used as weights of the edges between species (nodes) in the functional or co-occurrence networks.

For a functional or a co-occurrence network, modules were identified. Modules are distinct subsets of species that are mainly connected with each other, while they have far fewer connections to other species belonging to other modules (Girvan and Newman, 2002). For functional network, modules were considered to be equivalent to functional groups and identified from the functional similarity matrix using the ‘Nbclust’ function (with options distance = ‘euclidean’ and method = ‘ward.D’) in the R package ‘Nbclust’ (Charrad *et al*. 2014). This function calculates the best partitioning of the functional trait space into functional groups (=modules) comparing results when using 30 different indices. For co-occurrence networks, identification of modules was done via the Louvain optimization algorithm, which is recognized as one of the best algorithms for module detection (see Legras *et al*. 2019), using the R package ‘modMax’ (Schelling and Hui, 2015).

Finally, the congruence between the functional and the co-occurrence networks was assessed by an index of module diversity for each functional group of Gauzens *et al*. (2015), modified by Legras *et al*. (2019). This index was calculated for each group of species comprising a functional module, using the R code of Legras *et al*. (2019). It varies from zero to unity, being zero if all species of a functional module tend to belong to the same co-occurrence module and unity if all species of a functional module tend to belong to different co-occurrence modules. The index values of all functional modules of a network were averaged, resulting in the *Dg_M_* index. Subsequently, the value of the *Dg_M_* index for each compound, component, or infracommunity was compared with distribution of the *Dg_M_* values from 999 null models, in which the partition into functional modules and the number and size of co-occurrence modules were the same as in the original data, but species distribution among co-occurrence modules was random. The observed *Dg_M_* index was compared with the distribution of *Dg_M_* indices derived from null models. A significantly lower observed *Dg_M_* value than expected by chance (<5% of the null distribution) indicates the predominance of environmental/host-associated filtering, whereas a significantly higher value than expected by chance (>95% of the null distributions) suggests the effects of competition/limiting similarity (Legras *et al*. 2019). An observed *Dg_M_* value between 5% and 95% of the null *Dg_M_* distribution suggests either the effect of stochastic mechanisms or the lack of clear dominance of one of the deterministic processes (Legras *et al*. 2019). The R code for null modelling was taken from Legras *et al*. (2019). When the value of *Dg_M_* was calculated for a given community, only modules containing species from this community were retained in a functional network.

## Results

The best partitioning of the functional trait space resulted in the 21 functional groups (=functional modules) with average pairwise within-module similarity of 0.68 and between-module similarity of 0.59. Within-module functional similarity ranged from 0.70 to 0.93 (average 0.83 ± 0.04) for compound communities within biogeographic realms, from 0.70 to 0.82 (average 0.77 ± 0.01) for compound communities within geographic regions, from 0.62 to 0.95 (average 0.75 ± 0.01) for component communities, and from 0.62 to 0.98 (average 0.74 ± 0.02) for infracommunities (Tables 1–2, Tables S4–S5 in Supplementary Material, Appendix 1). The respective values of between-module similarity were 0.59–0.73 (average 0.65 ± 0.01), 0.60–0.65 (average 0.63 ± 0.01), 0.50–0.71 (average 0.62 ± 0.01) and 0.40–0.77 (average 0.60 ± 0.01) (Tables 1–2, Tables S4–S5 in Supplementary Material, Appendix 1).

**Table 1.** Congruence between functional (F) and co-occurrence (CoR: across regions; CoH: across host species) networks of compound flea communities in 6 biogeographic realms

Realm	Network	M	WS/BS	Modules	*Dg_M_*	*p*	Process
Afrotropics	F		0.70/0.61	13			
	CoR	0.57		8	0.77	0.04	EF
	CoH	0.14		4	0.52	<0.01	HF
Australasia	F		0.92/0.73	7			
	CoR	0.14		2	0.71	0.12	S
	CoH	0.32		12	0.46	0.07	EF
Indo-Malay	F		0.93/0.79	16			
	CoR	0.18		3	0.80	<0.01	EF
	CoH	0.21		3	0.87	0.10	S
Nearctic	F		0.81/0.65	17			
	CoR	0.40		2	0.90	0.04	EF
	CoH	0.28		10	0.79	0.02	EF
Neotropics	F		0.76/0.65	18			
	CoR	0.26		2	0.83	0.04	EF
	CoH	0.35		3	0.81	0.04	EF
Palearctic	F		0.87/0.66	17			
	CoR	0.46		3	0.90	<0.01	EF
	CoH	0.30		4	0.66	0.01	EF

M: modularity value (for co-occurrence networks), WS/BS: average within- and between-module similarity (for functional networks), Modules: number of detected modules, *Dg_M_*: index of congruence (see text for explanation). *p*: proportion of *Dg_M_* values from null models that are lower than the observed *Dg_M_*; Process: the most likely process affecting community assembly inferred from comparison of the observed and null *Dg_M_* values (EF, environmental filtering; HF, host-associated filtering; S, stochastic process(es) or no clear dominance of a deterministic process).

**Table 2.** Congruence between functional (F) and co-occurrence (CoL: across sampling sites; CoH: across host species) networks of compound flea communities in 7 regions

Region	Network	M	WS/BS	Modules	*Dg_M_*	*p*	Process
Mongolia	F		0.77/0.65	10			
	CoL	0.16		4	0.96	0.90	S
	CoH	0.06		4	0.82	0.04	HF
Northwest Argentina	F		0.75/0.65	8			
	CoL	0.22		6	0.91	0.61	S
	CoH	0.11		2	0.26	0.09	S
Patagonia	F		0.75/0.62	7			
	CoL	0.16		3	0.76	0.12	S
	CoH	0.07		3	0.87	0.74	S
Western Siberia	F	0.08	0.81/0.66	13			
	CoL	0.17		5	0.98	0.91	S
	CoH	0.06		4	0.95	0.59	S
Slovakia	F		0.82/0.68	6			
	CoL	0.06		3	0.49	<0.01	EF
	CoH	0.06		3	0.81	0.46	S
South Africa	F		0.70/0.60	5			
	CoL	0.23		4	0.44	0.18	S
	CoH	0.15		3	0.65	0.88	S
Tanzania	F		0.76/0.60	5			
	CoL	0.14		3	0.53	0.03	EF
	CoH	0.12		4	0.85	0.51	S

M: modularity value, WS/BS: average within- and between-module similarity (for functional networks), Modules: number of detected modules, *Dg_M_*: index of congruence (see text for explanation). *p*: proportion of *Dg_M_* values from null models that are lower than the observed *Dg_M_*; Process: the most likely process affecting community assembly inferred from comparison of the observed and null *Dg_M_* values (EF, environmental filtering; HF, host-associated filtering; S, stochastic process(es) or no clear dominance of a deterministic process).

Modularity in spatial co-occurrence networks ranged from 0.14 to 0.57 (average 0.34 ± 0.07) for compound communities within biogeographic realms, from 0.06 to 0.23 (average 0.16 ± 0.02) for compound communities within geographic regions, and from 0.01 to 0.28 (average 0.31 ± 0.01) for component communities (Tables 1–2, Tables S4–S5 in Supplementary Material, Appendix 1). Modularity values in networks of flea co-occurrences in host species (compound communities) or individuals (infracommunities) ranged from 0.14 to 0.35, from 0.06 to 0.15, and from 0.01 to 0.46 for compound communities at the scale of biogeographic realms or geographic regions and infracommunities, respectively (average 0.27 ± 0.03, 0.09 ± 0.01 and 0.20 ± 0.03, respectively) (Tables 1–2, Tables S4–S5 in Supplementary Material, Appendix 1).

**Figure 1.**
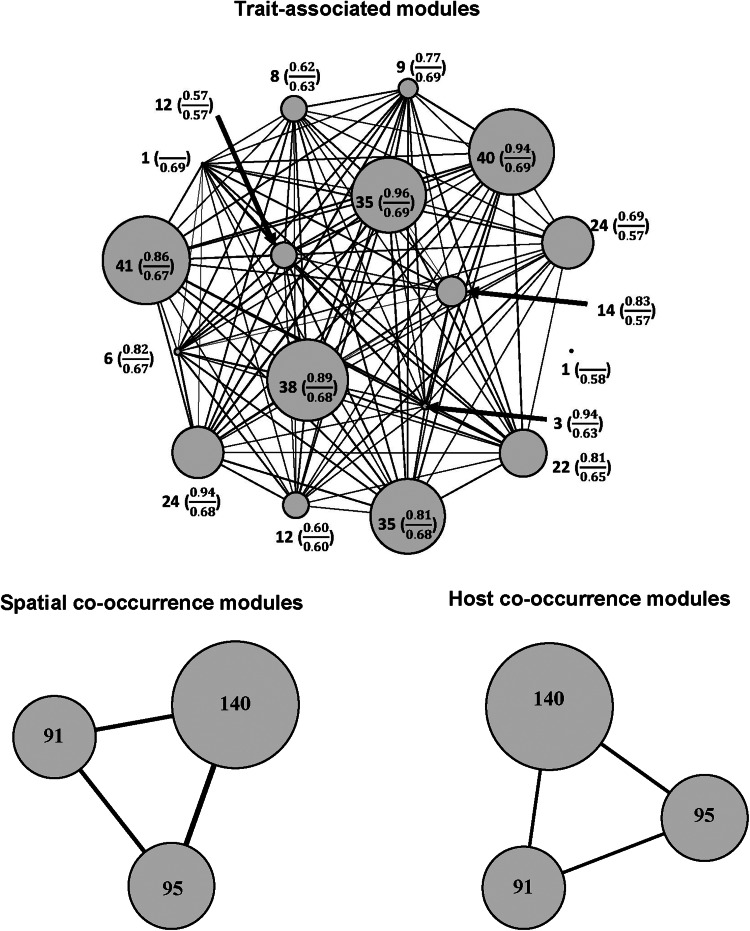
Modules based on trait similarity, spatial (across regions) co-occurrence similarity, and host species co-occurrence similarity for compound communities of fleas in the Palearctic. The number inside or near the circle is the number of species in the module. In trait-associated modules, the number in parentheses is average within-module similarity (above line) and between-module similarity (below line) between pairs of species. Edge width is proportional to average similarity between species belonging to the modules.

**Figure 2.**
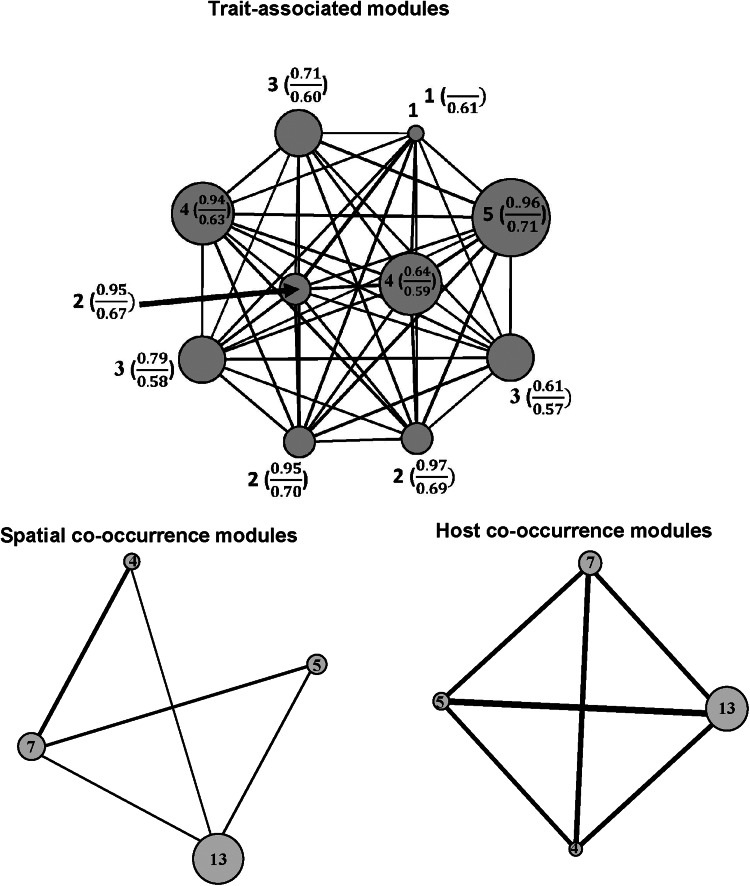
Modules based on trait similarity, spatial (across localities) co-occurrence similarity, and host species co-occurrence similarity for compound communities of fleas in Mongolia. The number inside or near the circle is the number of species in the module. In trait-associated modules, the number in parentheses is average within-module similarity (above line) and between-module similarity (below line) between pairs of species. Edge width is proportional to average similarity between species belonging to the modules.

The number of modules in functional networks ranged from 7 to 17 in realm-specific networks, from 5 to 13 in regional networks, from 2 to 8 in networks of component communities, and from 2 to 6 in infracommunities (Tables 1–2, Tables S5–S6 in Supplementary Material, Appendix 1). The Louvain algorithm detected 2–8 modules in spatial co-occurrence networks, and 2–12 modules in the networks of co-occurrence in host species/individuals (Tables 1–2, Tables S5–S6 in Supplementary Material, Appendix 1). In general, the number of detected modules in the co-occurrence networks was not associated with the number of species in a network, whereas the higher number of functional modules was detected in larger flea communities (see illustrative examples of module distribution in Figs 1–2 for compound communities and Figs. S1–S2 in Supplementary Material, Appendix 3 for component communities and infracommunities).

**Figure 3.**
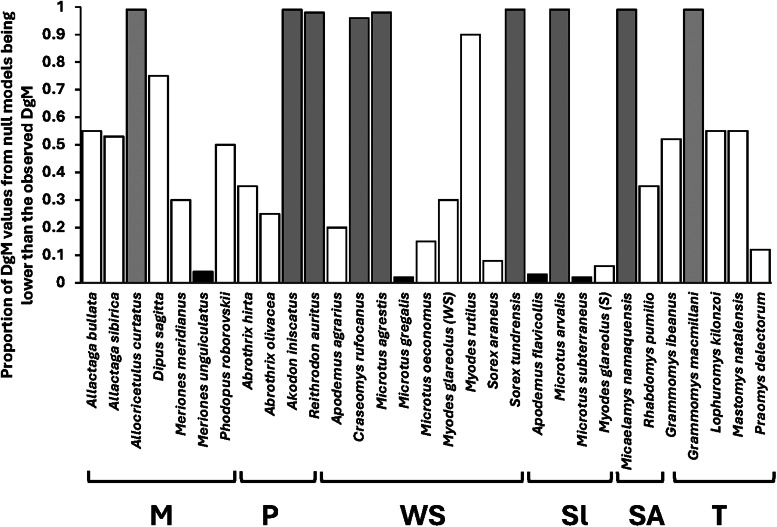
Proportions of *Dg_M_* values from null models that are lower than the observed *Dg_M_* calculated for assessing congruence between functional and co-occurrence networks in component communities of fleas harboured by 31 host species. The most likely process affecting community assembly, as inferred from the comparison of the observed and null *Dg_M_* values, is shown by bar colour (black: environmental filtering, grey: limiting similarity/competition, white: stochastic process(es) or no clear dominance of a deterministic process). M, Mongolia; P, Patagonia, WS, Western Siberia, Sl, Slovakia; SA, South Africa; T, Tanzania.

**Figure 4.**
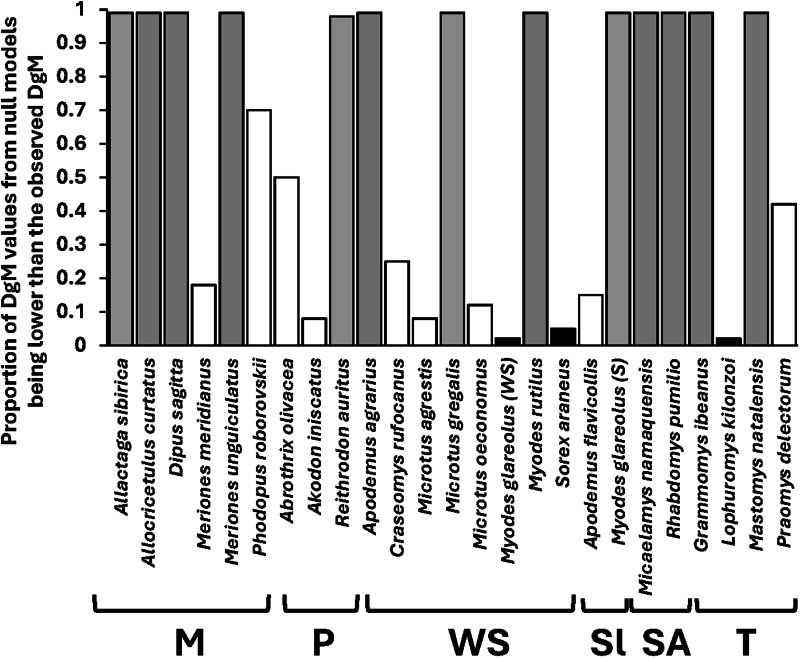
Proportions of *Dg_M_* values from null models that are lower than the observed *Dg_M_* calculated for assessing congruence between functional and co-occurrence networks in infracommunities of fleas harboured by 25 host species. The most likely process affecting community assembly as inferred from the comparison of the observed and null *Dg_M_* values is shown by bar colour (black: host-associated filtering, grey: limiting similarity/competition, white: stochastic process(es) or no clear dominance of a deterministic process). M, Mongolia; P, Patagonia; WS, Western Siberia; Sl, Slovakia; SA, South Africa; T; Tanzania.

Comparisons of the observed *Dg_M_* values with the distributions of those obtained from null modelling demonstrated that functional and spatial or host-associated co-occurrence networks of fleas in compound communities within biogeographic realms were consistently congruent, except for spatial co-occurrence in Australasia and host-associated co-occurrence in Indo-Malay, and suggested that these communities (both across regions and across host species) were assembled via environmental or host-associated filtering, respectively (Table 1). In contrast, functional and spatial/host-associated co-occurrence networks, at the scale of regional compound communities (i.e. across localities), were mostly incongruent (except the spatial co-occurrence network in Slovakia and Tanzania and the host-associated co-occurrence network in Mongolia, both suggesting environmental/host-associated filtering) (Table 2). The incongruence between the networks indicated either stochastic processes or the lack of dominance of any deterministic process. Analyses of congruence between functional and either spatial (for component communities) or host-associated (for infracommunities) co-occurrence networks demonstrated that assembly rules in these communities varied among the host species harbouring them. In 18 of 31 component communities, stochastic processes in their assembly were indicated, whereas environmental filtering seemed to dominate in 4, and limiting similarity/competition in 9 communities (Fig. 3, see detailed results in Table S4 in Supplementary Material, Appendix 1). Stochastic assembly processes dominated in infracommunities harboured by 9 of 25 host species, limiting similarity/competition in infracommunities of 13 host species, and host-associated filtering in the infracommunities of 3 hosts (*Microtus oeconomus*, *Sorex araneus* and *Lophuromys kilonzoi*) (Fig. 4, see detailed results in Table S5 in Supplementary Material, Appendix 1).

## Discussion

We found substantial differences in the patterns of species co-occurrence and the processes dominating flea community assembly at different hierarchical scales. Moreover, these patterns and processes were consistent at some scales and contingent at other scales. In particular, regional compound communities within biogeographic realms were characterized by positive species associations across both regions and host species, suggesting the important role of environmental or host-associated filtering. On the contrary, local compound communities within a relatively homogeneous region demonstrated random species associations and, thus, indicating the role of stochastic factors. In component and infracommunities, patterns of species associations and, consequently, presumed assembly rules varied greatly among communities harboured by different host species. This supported the results of some, but not other, earlier studies on co-occurrence patterns in flea communities (Krasnov *et al*. 2010, 2011, 2015 vs Krasnov *et al*. 2006*a*, 2014; Vinarski *et al*. 2020; Gibert *et al*. 2021, respectively).

The mechanism behind the aggregative pattern of flea co-occurrences in regional compound communities within a biogeographic realm, coupled with the trait similarity of co-occurring species, is associated with similar environmental and host-associated preferences of phylogenetically close flea species possessing similar traits (Krasnov *et al*. 2015). The environmental factors determining flea preferences are, first and foremost, air temperature and relative humidity (to which fleas are highly sensitive; Margalit and Shulov, 1972), as well as vegetation structure (see review in Krasnov, 2008). These factors affect the microclimate in hosts’ burrows where pre-imagoes develop, and adult fleas spend substantial time. An additional environmental factor is soil structure since soil particles are used by flea larvae for cocoon construction (Marshall, 1981). Particles of different soil types likely differ in their water-absorption abilities, while active water vapour uptake is highly important for cocooned fleas (Rudolph and Knulle, 1982). In Tanzania, flea abundance was found to be affected by soil properties such as exchangeable calcium and magnesium, phosphorus availability and base saturation (Meliyo *et al*. 2014). Obviously, environmental differences between regions of the same biogeographic realm are sharp, resulting in further classification into biomes and/or ecoregions (e.g. Olson *et al*. 2001). Furthermore, regions within a realm differ not only in their abiotic environment but also in the species composition of small mammals that fleas can utilize as their hosts. The process of host selection by parasites is largely determined by the complementarity of co-evolving host and parasite traits (McQuaid and Britton, 2013; dos Santos Cardoso *et al*. 2021; see Krasnov *et al*. 2016 for fleas). Host–parasite co-evolution consists of parasite adaptation to extract resources from their hosts, as well as to evade or overcome their defences, and reciprocal host counter-adaptation to avoid or tolerate infection (e.g. Buckingham and Ashby, 2022). Complementarity or matching between parasite and host traits may result in establishing novel parasite–host associations when a host or a parasite invades new areas (e.g. Schatz and Park, 2021). Complementary parasite–host traits cause host species composition to act as a filter for assembly of a regional parasite community. The reason for not detecting environmental or host-associated filtering processes as assembly mechanisms, for compound flea communities in the Australasian and Indo-Malayan realms, respectively, could be the much poorer knowledge of either geographic distribution of fleas or host–flea associations in these than in other realms. For example, only 2 flea species parasitic on small mammals were recorded in Northern Territories of Australia (Dunnet and Mardon, 1974). Completely different types of analyses, carried out on almost the same data as in the present study, suggested the predominance of the dispersal-based mechanism of flea compound community assembly within biogeographic realms realized via host dispersal (Gibert *et al*. 2021). However, these analyses did not consider trait difference/similarity between species and were based on a pairwise comparison of communities between continental sections/landscapes rather than between regions.

In contrast to regional compound communities within a biogeographic realm, functional and co-occurrence networks, at the scale of local communities within a region, were mostly incongruent. On the one hand, this could indicate stochastic birth/death processes of community assembly, resulting in ‘neutral’ communities that are primarily shaped by population dynamics and/or dispersal limitations (Hubbell, 2001). On the other hand, counterbalancing of environmental filtering and limiting similarity/competition may lead to a pattern ostensibly resembling neutrality (Purves and Pacala, 2005). The latter seems to be more likely because at least some, albeit only two, compound communities (Slovakian and Mongolian) suggested the acting of deterministic processes. The lack of a clear indication of any deterministic process at this scale may also be associated with relative environmental homogeneity within a region, so that environmental differences, as well as differences in host species composition, were not sharp enough for environmental/host-associated filtering to be detected (but see McNew *et al*. 2021). Another reason for the lack of detection of deterministic processes at this scale is that compound communities across localities within a region are represented by the set of communities harboured by different host species (i.e. component communities). Processes affecting these communities’ assembly varied both between regions and between host species. Variation in the degree of congruence between flea functional and co-occurrence networks and flea community assembly processes could stem from ecological and/or physiological differences between hosts, such as mobility patterns, burrow/nest structure, population density and immunocompetence as well as from differences in relative numbers of flea species with a certain set of traits. For example, if only a small number of flea species possess certain traits allowing them to overcome host defences and exploit strongly immunocompetent hosts (Møller *et al*. 2005) (which the remaining fleas from the same community cannot), then the associations between these and the remaining fleas would be negative, leading to a pattern superficially resembling the effect of limiting similarity/competition. If, however, the number of flea species capable of overcoming host defences is high, then positive associations between these species would be found, and a process resembling environmental filtering would be detected. Therefore, the manifestation of a given assembly rule in parasite component communities might be the result of interplay between parasite traits, host traits and parasite community composition. It is well known that the pattern of species association within a metacommunity varies between pairs or groups of species, from positive to random to negative (Sfenthourakis *et al*. 2006; D'Amen *et al*. 2018). The conclusion regarding the predominant pattern of species associations and, consequently, the rule of community assembly would depend on the relative number of species pairs/groups with a given association pattern.

Functional and co-occurrence networks in flea infracommunities were congruent in some, but incongruent in other, host species. Among 16 hosts in the infracommunities in which between-network congruence was found, a limiting similarity/competition process was detected in 13 hosts and environmental filtering in 3 hosts. The occurrence of competition among fleas exploiting the same host individual contradicts studies on the patterns of flea co-occurrences (Krasnov *et al*. 2006*a*, 2010) but supports experimental and observational studies that reported possible interspecific competition (Day and Benton, 1980; Krasnov *et al*. 2005; Surkova *et al*. 2018). Interestingly, evidence for the interspecific flea competition found in experimental studies has suggested that it (a) is asymmetric and (b) occurs among larvae rather than imago fleas. Intraspecific larval competition may or may not be further manifested in the pattern of imago co-occurrence. Moreover, parasite infracommunities are ephemeral not only from the perspective of a host individual's lifespan (Poulin, 2007) but, in the case of fleas, from the fact that infracommunities’ presence and composition are temporally variable and may change daily (Krasnov *et al*. 2006*b*). This explains the variation in the detection of deterministic and stochastic processes in infracommunity assembly.

In conclusion, assembly processes in parasite communities appeared to be scale-dependent, with different mechanisms acting at different scales (see also McNew *et al*. 2021). The main reason for this is that community composition at the largest scale is predominantly affected by evolutionary, historical and biogeographical processes, whereas community composition at smaller scales is mainly determined by ecological and demographic processes. The approach combining species traits and patterns of groupwise, rather than pairwise, co-occurrence improves the reliability of detection and understanding of processes of community assembly. Regarding parasites, this approach could also be helpful from the perspective of the dynamics of vector-borne diseases (Makundi *et al*. 2015).

## Supporting information

Krasnov et al. supplementary material Krasnov et al. supplementary material

## Data Availability

Raw data on flea and host species at the scale of biogeographic realms are contained in the sources cited in Krasnov *et al*. (2022). The remaining data can be obtained from the corresponding author upon request.

## Supplementary material

The supplementary material for this article can be found at https://doi.org/10.1017/S0031182024000969.
