# Supplementary material for: Congruence between co-occurrence and trait-based networks is scale-dependent: a case study with flea parasites of small mammalian hosts
Source: Parasitology. 2024 Oct 8;151(8):853–63. doi: 10.1017/S0031182024000969 (PMC11578890; doi:10.1017/S0031182024000969)
Supplement: Krasnov et al. supplementary material [file S0031182024000969sup001.docx]

**Appendix 1. Supplementary tables.**

**Table S1.** Lists of flea and host species used in the analyses at the scale of compound flea communities across localities within a region.

| Region | List of flea species | List of host species |
| --- | --- | --- |
| Mongolia | *Amphipsylla longispina*  *Amphipsylla primaris*  *Ampphipsylla vinogradovi*  *Citellophilus altaicus*  *Citellophilus sungaris*  *Coptopsylla lamelifer*  *Ctenophthalmus arvalis*  *Echidnophaga oshanini*  *Echidnophaga tiscadaea*  *Frontopsylla elata*  *Frontopsylla hetera*  *Frotopsylla luculenta*  *Frontopsylla wagneri*  *Mesopsylla eucta*  *Mesopsylla hebes*  *Neopsylla acanthina*  *Neopsylla bidentata*  *Neopsylla pleskei*  *Nosopsyllus laeviceps*  *Ochotonobis hirticrus*  *Ophthalmopsylla kiritschenkovi*  *Ophthalmopsylla kukuschkini*  *Ophthalmopsylla praefecta*  *Ophthalmopsylla volgensis*  *Paramonopsyllus scalonae*  *Paradoxopsyllus scorodumovi*  *Pectinoctenus pavlovskii*  *Xenopsylla conformis*  *Xenopsylla skrjabini* | *Allactaga balikunica*  *Allactaga bullata*  *Allactaga elater*  *Allactaga sibirica*  *Allocricetulus curtatus*  *Alticola semicanus*  *Apodemus peninsulae*  *Cardiocranius paradoxus*  *Cricetulus longicaudatus*  *Cricetulus migratorius*  *Dipus sagitta*  *Euchoreutes naso*  *Hemiechinus auritus*  *Lagurus lagurus*  *Lasiopodomys brandtii*  *Marmota sibirica*  *Meriones meridianus*  *Merriones unguiculatus*  *Ochotona daurica*  *Ochotona pallasii*  *Phodopus campbelli*  *Phodopus roborovskii*  *Pygeretmus pumilio*  *Rhombomys opimus*  *Salpingotus crassicauda*  *Spermophilus alashanicus*  *Spermophilus erythrourus*  *Stylodipus andrewsi*  *Urocitellus undulatus* |
| Northwest Argentina | *Adoratopsylla intermedia*  *Agastopsylla nylota*  *Cleopsylla barquezi*  *Craneopsylla minerva*  *Ctenidiosomus austrinus*  *Delostichus talis*  *Ectinorus monicadiazae*  *Ectinorus sp.*  *Hectopsylla gracilis*  *Neotyphloceras chilensis*  *Neotyphloceras crassispina*  *Plocopsylla chicoanaensis*  *Plocopsylla inti*  *Plocopsylla sp.*  *Polygenis acodontis*  *Polygenis byturus*  *Polygenis pradoi*  *Polygenis puelche*  *Polygenis sp.*  *Polygenis tripus*  *Tetrapsyllus bleptus*  *Tetrapsyllus spegazzinii*  *Tiamastus cavicola*  *Tiamastus palpalis* | *Abrothrix andina*  *Akodon albiventer*  *Akodon budini*  *Akodon caenosus*  *Akodon dolores*  *Akodon glaucinus*  *Akodon lutescens*  *Akodon puer*  *Akodon simulator*  *Akodon* sp.  *Akodon spegazzinii*  *Akodon tartareus*  *Akodon tucumanensis*  *Calomys boliviae*  *Calomys callosus*  *Calomys musculinus*  *Calomys* sp.  *Calomys venustus*  *Ctenomys* sp.  *Eligmodontia bolsonensis*  *Eligmodontia puerulus*  *Eligmodontia* sp.  *Graomys chacoensis*  *Graomys griseoflavus*  *Lutreolina massoia*  *Microcavia maenas*  *Necromys lactens*  *Neotomys ebriosus*  *Oligoryzomys brendae*  *Oligoryzomys flavescens*  *Oxymycterus paramensis*  *Phyllotis caprinus*  *Phyllotis osilae*  *Phyllotis xanthopygus*  *Tapecomys primus*  *Thylamys sponsorius* |
| Patagonia | *Agastopsylla boxi*  *Craneopsylla minerva*  *Ectinorus galeanus*  *Ectinorus hapalus*  *Ectinorus ixanus*  *Ectinorus levipes*  *Ectinorus martini*  *Ectinorus onychius*  *Hectopsylla gracilis*  *Neotyphloceras crackensis*  *Neotyphloceras pardinasii*  *Plocopsylla lewisi*  *Plocopsylla silewi*  *Plocopsylla wilesi*  *Polygenis platensis*  *Polygenis rimatus*  *Sphinctopsylla ares*  *Tetrapsyllus bleptus*  *Tetrapsyllus rhombus*  *Tetrapsyllus tantillus*  *Tiamastus callens*  *Tiarapsylla argentina* | *Abrothrix hirta*  *Abrothrix olivacea*  *Akodon dolores*  *Akodon iniscatus*  *Calomys musculinus*  *Chelemys macronyx*  *Ctenomys* sp.  *Eligmodontia morgani*  *Eligmodontia typus*  *Euneomys chinchilloides*  *Euneomys petersoni*  *Graomys griseoflavus*  *Loxodontomys micropus*  *Microcavia australis*  *Phyllotis xanthopygus*  *Reithrodon auritus*  *Thylamys pallidior* |
| Western Siberia | *Amalareus penicilliger*  *Amphipsulla kuznetzovi*  *Amphipsylla primaris*  *Amphipsylla rossica*  *Amphipsylla sibirica*  *Amphalius runatus*  *Catallagia dacenkoi*  *Catallagia ioffi*  *Ceratophylus indages*  *Citellophilus tesquorum*  *Corrodopsylla birulai*  *Ctenophthalmus arvalis*  *Ctenophthalmus asssimilis*  *Ctenophthalmus breviatus*  *Ctenophthalmus uncinatus*  *Ctenophthalmus wagneri*  *Doratopsylla dasycnema*  *Frotopsylla elata*  *Hystrichopsylla talpae*  *Leptopsylla segnis*  *Megabothris calcarifer*  *Megabothris rectangulatus*  *Megabothris turbidus*  *Megabothris walkeri*  *Neopsylla acanthina*  *Neopsylla mana*  *Neopsylla pleskei*  *Palaeopsylla soricis*  *Pectinoctenus pavlovskii*  *Peromyscopsylla bidentata*  *Peromyscopsylla silvatica*  *Rhadinopsylla altaica*  *Rhadinopsylla integella* | *Alticola strelzovi*  *Apodemus agrarius*  *Apodemus peninsulae*  *Apodemus speciosus*  *Apodemus uralensis*  *Arvicola amphibius*  *Craseomys rufocanus*  *Cricetus cricetus*  *Dicrostonyx torquatus*  *Eutamias sibiricus*  *Lagurus lagurus*  *Lemmmus sibiricus*  *Micromys minutus*  *Microtus agrestis*  *Microtus arvalis*  *Microtus gregalis*  *Microtus middendorffii*  *Microtus oeconomus*  *Mus musculus*  *Myodes glareolus*  *Myodes rutilus*  *Neomys fodiens*  *Ochotona alpina*  *Phodopus sungorus*  *Sicista betulina*  *Sorex araneus*  *Sorex caecutiens*  *Sorex daphaenodon*  *Sorex isodon*  *Sorex minutus*  *Sorex roboratus*  *Sorex tundrensis*  *Talpa altaica* |
| Slovakia | *Amalaraeus arvicolae*  *Amalaraeus penicilliger*  *Ceratophyllus sciurorum*  *Ctenophthalmus agyrtes*  *Ctenophthalmus assimilis*  *Ctenophthalmus bisoctodentatus*  *Ctenophthalmus obtusus*  *Ctenophthalmus solutus*  *Ctenophthalmus uncinatus*  *Doratopsylla dasycnema*  *Hystrichopsylla orientalis*  *Hystrichopsylla talpae*  *Leptopsylla segnis*  *Megabothris turbidus*  *Nosopsyllus fasciatus*  *Peromyscopsylla bidentata*  *Peromyscopsylla silvatica*  *Palaeopsylla similis*  *Palaeopsylla soricis*  *Rhadinopsylla integella*  *Rhadinopsylla penthacanta* | *Apodemus agrarius*  *Apodemus flavicollis*  *Apodemus sylvaticus*  *Apodemus uralensis*  *Arvicola amphibius*  *Crocidura leucodon*  *Glis glis*  *Micromys minutus*  *Microtus agrestis*  *Microtus arvalis*  *Microtus subterraneus*  *Muscardinus avellanarius*  *Mus musculus*  *Myodes glareolus*  *Neomys anomalus*  *Neomys fodiens*  *Rattus norvegicus*  *Sicista betulina*  *Sorex alpinus*  *Sorex araneus*  *Sorex minutus*  *Spermophilus citellus*  *Talpa europaea* |
| South Africa | *Chiastopsylla capensis*  *Chiastopsylla carus*  *Chiastopsylla coraxis*  *Chiastopsylla godfreyi*  *Chiastopsylla mulleri*  *Chiastopsylla nama*  *Chiastopsylla octavii*  *Chiastopsylla pitchfordi*  *Chiastopsylla quadrisetis*  *Chiastopsylla rossi*  *Ctenophthalmus calceatus*  *Ctenophthalmus natalensis*  *Demeillionia granti*  *Dinopsyllus ellobius*  *Dinopsyllus lypusus*  *Dinopsyllus tenax*  *Epirimia aganippes*  *Hypsophthalmus temporis*  *Listropsylla agrippinae*  *Listropsylla aricinae*  *Listropsylla dorippae*  *Praopsylla powelli*  *Xenopsylla brasiliensis*  *Xenopsylla eridos*  *Xenopsylla piriei*  *Xenopsylla trifaria* | *Crocidura* sp.  *Desmodillus auricularis*  *Elephantulus edwwardii*  *Gerbillurus paeba*  *Mastomys natalensis*  *Micaelamys namaquensis*  *Mus minutoides*  *Mus musculus*  *Myotomys unisulcatus*  *Otomys irroratus*  *Parotomys brantsii*  *Rhabdomys pumilio*  *Myosorex* sp. |
| Tanzania | *Afristivalius torvus*  *Chiastopsylla rossi*  *Ctenophthalmus calceatus*  *Ctenophthalmus cophurus*  *Ctenophthalmus eximius*  *Ctenophthalmus hopkinsi*  *Ctenophthalmus kemmelberg*  *Ctenophthalmus leptodactylus*  *Ctenophthalmus teucqae*  *Dinopsyllus grypurus*  *Dinopsyllus longifrons*  *Dinopsyllus lypusus*  *Dinopsyllus pringlei*  *Dinopsyllus titan*  *Hypsophthalmus campestris*  *Leptopsylla aethiopica*  *Lybiastus duratus*  *Nosopsyllus incisus*  *Xenopsylla brasiliensis*  *Xenopsylla sarodes*  *Xiphiopsylla hyparetes* | *Aethomys chrysophilus*  *Arvicanthis* sp.  *Cricetomys gambianus*  *Crocidura hildegardeae*  *Crocidura poensis*  *Crocidura* sp.  *Dendromus nyikae*  *Grammomys ibeanus*  *Grammomys macmillani*  *Grammomys* sp.  *Hylomyscus acrimontensis*  *Lemniscomys rosalia*  *Lophuromys aquilus*  *Mastomys natalensis*  *Otomys sp.*  *Paraxerus vexillarius*  *Praomys delectorum*  *Rattus rattus* |

**Table S2.** Host species used in the analyses at the scale of component flea communities.

| Region | Host species | Number of sampling sites | Flea species richness |
| --- | --- | --- | --- |
| Mongolia | *Allactaga bullata* | 36 | 12 |
|  | *Allactaga sibirica* | 52 | 15 |
|  | *Allocricetulus curtatus* | 16 | 10 |
|  | *Dipus sagitta* | 40 | 11 |
|  | *Meriones meridianus* | 38 | 10 |
|  | *Meriones unguiculatus* | 17 | 9 |
|  | *Phodopus roborovskii* | 22 | 10 |
| Patagonia | *Abrothrix hirta* | 5 | 8 |
|  | *Abrothrix olivacea* | 13 | 12 |
|  | *Akodon iniscatus* | 9 | 8 |
|  | *Reithrodon auritus* | 7 | 8 |
| Western Siberia | *Apodemus agrarius* | 15 | 11 |
|  | *Craseomys rufocanus* | 18 | 12 |
|  | *Microtus agrestis* | 18 | 13 |
|  | *Microtus gregalis* | 17 | 13 |
|  | *Microtus oeconomus* | 30 | 17 |
|  | *Myodes glareolus* | 22 | 16 |
|  | *Myodes rutilus* | 42 | 23 |
|  | *Sorex araneus* | 29 | 12 |
|  | *Sorex tundrensis* | 10 | 9 |
| Slovakia | *Apodemus flavicollis* | 13 | 12 |
|  | *Microtus arvalis* | 10 | 8 |
|  | *Microtus subterraneus* | 11 | 8 |
|  | *Myodes glareolus* | 13 | 13 |
| South Africa | *Micaelamys namaquensis* | 5 | 9 |
|  | *Rhabdomys pumilio* | 36 | 15 |
| Tanzania | *Grammomys ibeanus* | 10 | 12 |
|  | *Grammomys macmillani* | 7 | 8 |
|  | *Lophuromys kilonzoi* | 10 | 14 |
|  | *Mastomys natalensis* | 11 | 9 |
|  | *Praomys delectorum* | 10 | 12 |

**Table S3.** Host species used in the analyses at the scale of flea infracommunities.

| Region | Host species | Number of host individuals | Flea species richness |
| --- | --- | --- | --- |
| Mongolia | *Allactaga sibirica* | 10 | 7 |
|  | *Allocricetulus curtatus* | 5 | 8 |
|  | *Dipus sagitta* | 11 | 6 |
|  | *Meriones meridianus* | 12 | 8 |
|  | *Meriones unguiculatus* | 9 | 6 |
|  | *Phodopus roborovskii* | 16 | 10 |
| Patagonia | *Abrothrix olivacea* | 16 | 9 |
|  | *Akodon iniscatus* | 17 | 7 |
|  | *Reithrodon auritus* | 22 | 10 |
| Western Siberia | *Apodemus agrarius* | 12 | 8 |
|  | *Craseomys rufocanus* | 82 | 12 |
|  | *Microtus agrestis* | 33 | 9 |
|  | *Microtus gregalis* | 44 | 9 |
|  | *Microtus oeconomus* | 84 | 13 |
|  | *Myodes glareolus* | 23 | 10 |
|  | *Myodes rutilus* | 124 | 13 |
|  | *Sorex araneus* | 113 | 10 |
| Slovakia | *Apodemus flavicollis* | 199 | 9 |
|  | *Myodes glareolus* | 37 | 7 |
| South Africa | *Micaelamys namaquensis* | 18 | 6 |
|  | *Rhabdomys pumilio* | 33 | 6 |
| Tanzania | *Grammomys ibeanus* | 9 | 6 |
|  | *Lophuromys kilonzoi* | 25 | 11 |
|  | *Mastomys natalensis* | 24 | 6 |
|  | *Praomys delectorum* | 32 | 8 |

**Table S4.** Congruence between functional (F) and co-occurrence (Co) networks of component flea communities in 31 host species from six regions. M: modularity value, WS/BS: average within- and between-module similarity (for functional networks), Modules: number of detected modules, *Dg_M_*: index of congruence (see text for explanation). *p*: proportion of *Dg_M_* values from null models that are lower than the observed *Dg_M_*; Process: the most likely process affecting community assembly inferred from comparison of the observed and null *Dg_M_* values (HF: host-associated filtering, LS: limiting similarity, S: stochastic).

| Host | Network | M | WS/BS | Modules | *Dg_M_* | *p* | Process |
| --- | --- | --- | --- | --- | --- | --- | --- |
| *Allactaga bullata* | F |  | 0.75/0.63 | 5 |  |  |  |
|  | Co | 0.19 |  | 4 | 0.92 | 0.69 | S |
| *Allactaga sibirica* | F |  | 0.72/0.64 | 6 |  |  |  |
|  | Co | 0.19 |  | 4 | 0.88 | 0.52 | S |
| *Allocricetulus curtatus* | F |  | 0.75/0.63 | 5 |  |  |  |
|  | Co | 0.01 |  | 3 | 0.99 | 0.99 | LS |
| *Dipus sagitta* | F |  | 0.78/0.54 | 4 |  |  |  |
|  | Co | 0.25 |  | 3 | 0.85 | 0.75 | S |
| *Meriones meridianus* | F |  | 0.69/0.58 | 5 |  |  |  |
|  | Co | 0.01 |  | 2 | 0.85 | 0.30 | S |
| *Meriones unguiculatus* | F |  | 0.63/0.59 | 3 |  |  |  |
|  | Co | 0.13 |  | 3 | 0.16 | 0.04 | EF |
| *Phodopus roborovskii* | F |  | 0.75/0.63 | 4 |  |  |  |
|  | Co | 0.1 |  | 3 | 0.84 | 0.48 | S |
| *Abrothrix hirta* | F |  | 0.71/0.55 | 3 |  |  |  |
|  | Co | 0.12 |  | 2 | 0.73 | 0.32 | S |
| *Abrothrix olivacea* | F |  | 0.74/0.63 | 3 |  |  |  |
|  | Co | 0.07 |  | 3 | 0.63 | 0.22 | S |
| *Akodon iniscatus* | F |  | 0.85/0.58 | 4 |  |  |  |
|  | Co | 0.14 |  | 2 | 0.99 | 0.99 | LS |
| *Reithrodon auritus* | F |  | 0.77/0.56 | 5 |  |  |  |
|  | Co | 0.07 |  | 2 | 0.92 | 0.98 | LS |
| *Apodemus agrarius* | F |  | 0.72/0.60 | 4 |  |  |  |
|  | Co | 0.13 |  | 3 | 0.78 | 0.18 | S |
| *Craseomys rufocanus* | F |  | 0.73/0.61 | 5 |  |  |  |
|  | Co | 0.11 |  | 3 | 0.97 | 0.99 | LS |
| *Microtus agrestis* | F |  | 0.74/0.67 | 4 |  |  |  |
|  | Co | 0.09 |  | 3 | 0.97 | 0.98 | LS |
| *Microtus gregalis* | F |  | 0.73/0.65 | 4 |  |  |  |
|  | Co | 0.16 |  | 3 | 0.55 | 0.02 | EF |
| *Microtus oeconomus* | F |  | 0.76/0.69 | 8 |  |  |  |
|  | Co | 0.12 |  | 4 | 0.86 | 0.12 | S |
| *Myodes glareolus* (Siberia) | F |  | 0.75/0.69 | 4 |  |  |  |
|  | Co | 0.09 |  | 4 | 0.96 | 0.25 | S |
| *Myodes rutilus* | F |  | 0.76/0.69 | 6 |  |  |  |
|  | Co | 0.12 |  | 4 | 0.97 | 0.90 | S |
| *Sorex araneus* | F |  | 0.69/0.65 | 5 |  |  |  |
|  | Co | 0.09 |  | 2 | 0.83 | 0.07 | S |
| *Sorex tundrensis* | F |  | 0.72/0.69 | 4 |  |  |  |
|  | Co | 0.17 |  | 2 | 0.82 | 0.99 | LS |
| *Apodemus flavicollis* | F |  | 0.77/0.67 | 6 |  |  |  |
|  | Co | 0.02 |  | 3 | 0.57 | 0.03 | EF |
| *Microtus arvalis* | F |  | 0.88/0.68 | 3 |  |  |  |
|  | Co | 0.07 |  | 2 | 0.90 | 0.99 | LS |
| *Microtus subterraneus* | F |  | 0.95/0.63 | 4 |  |  |  |
|  | Co | 0.1 |  | 2 | 0.46 | 0.02 | EF |
| *Myodes glareolus* (Slovakia) | F |  | 0.85/0.71 | 5 |  |  |  |
|  | Co | 0.08 |  | 3 | 0.69 | 0.25 | S |
| *Micaelamys namaquensis* | F |  | 0.67/0.55 | 2 |  |  |  |
|  | Co | 0.22 |  | 2 | 0.55 | 0.98 | LS |
| *Rhabdomys pumilio* | F |  | 0.62/0.50 | 4 |  |  |  |
|  | Co | 0.20 |  | 4 | 0.33 | 0.35 | S |
| *Grammomys ibeanus* | F |  | 0.82/0.61 | 5 |  |  |  |
|  | Co | 0.28 |  | 3 | 0.85 | 0.55 | S |
| *Grammomys macmillani* | F |  | 0.69/0.58 | 4 |  |  |  |
|  | Co | 0.18 |  | 3 | 0.99 | 0.99 | LS |
| *Lophuromys kilonzoi* | F |  | 0.75/0.62 | 5 |  |  |  |
|  | Co | 0.15 |  | 2 | 0.75 | 0.12 | S |
| *Mastomys natalensis* | F |  | 0.80/0.59 | 5 |  |  |  |
|  | Co | 0.19 |  | 3 | 0.94 | 0.52 | S |
| *Praomys delectorum* | F |  | 0.82/0.64 | 4 |  |  |  |
|  | Co | 0.03 |  | 3 | 0.65 | 0.12 | S |

**Table S5.** Congruence between functional (F) and co-occurrence (Co) networks of flea infracommunities in 25 host species from six regions. M: modularity value, WS/BS: average within- and between-module similarity (for functional networks), Modules: number of detected modules, *Dg_M_*: index of congruence (see text for explanation). *p*: proportion of *Dg_M_* values from null models that are lower than the observed *Dg_M_*; Process: the most likely process affecting community assembly inferred from comparison of the observed and null *Dg_M_* values (EF: environmental filtering, LS: limiting similarity, S: stochastic).

| Host | Network | M | WS/BS | Modules | *Dg_M_* | *p* | Process |
| --- | --- | --- | --- | --- | --- | --- | --- |
| *Allocricetulus curtatus* | F |  | 0.87/0.70 | 5 |  |  |  |
|  | Co | 0.23 |  | 2 | 0.95 | 0.99 | LS |
| *Allactaga sibirica* | F |  | 0.98/0.77 | 4 |  |  |  |
|  | Co | 0.33 |  | 3 | 0.99 | 0.99 | LS |
| *Dipus sagitta* | F |  | 0.77/0.40 | 2 |  |  |  |
|  | Co | 0.04 |  | 3 | 0.66 | 0.99 | LS |
| *Meriones meridianus* | F |  | 0.65/0.56 | 4 |  |  |  |
|  | Co | 0.11 |  | 3 | 0.77 | 0.18 | S |
| *Meriones unguiculatus* | F |  | 0.75/0.46 | 2 |  |  |  |
|  | Co | 0.22 |  | 2 | 0.83 | 0.99 | LS |
| *Phodopus roborovskii* | F |  | 0.75/0.62 | 4 |  |  |  |
|  | Co | 0.35 |  | 3 | 0.93 | 0.70 | S |
| *Abrothrix olivacea* | F |  | 0.76/0.63 | 3 |  |  |  |
|  | Co | 0.08 |  | 3 | 0.80 | 0.47 | S |
| *Akodon iniscatus* | F |  | 0.85/0.61 | 4 |  |  |  |
|  | Co | 0.18 |  | 3 | 0.66 | 0.08 | S |
| *Reithrodon auritus* | F |  | 0.77/0.56 | 5 |  |  |  |
|  | Co | 0.15 |  | 3 | 0.94 | 0.98 | LS |
| *Apodemus agrarius* | F |  | 0.84/0.56 | 2 |  |  |  |
|  | Co | 0.28 |  | 3 | 0.77 | 0.98 | LS |
| *Craseomys rufocanus* | F |  | 0.73/0.61 | 5 |  |  |  |
|  | Co | 0.09 |  | 5 | 0.80 | 0.25 | S |
| *Microtus agrestis* | F |  | 0.70/0.65 | 4 |  |  |  |
|  | Co | 0.07 |  | 2 | 0.80 | 0.08 | S |
| *Microtus gregalis* | F |  | 0.75/0.67 | 4 |  |  |  |
|  | Co | 0.27 |  | 3 | 0.99 | 0.99 | LS |
| *Microtus oeconomus* | F |  | 0.71/0.61 | 6 |  |  |  |
|  | Co | 0.10 |  | 3 | 0.75 | 0.01 | HF |
| *Myodes glareolus* (Siberia) | F |  | 0.68/0.62 | 4 |  |  |  |
|  | Co | 0.35 |  | 4 | 0.37 | 0.15 | S |
| *Myodes rutilus* | F |  | 0.72/0.62 | 2 |  |  |  |
|  | Co | 0.06 |  | 3 | 0.64 | 0.99 | LS |
| *Sorex araneus* | F |  | 0.67/0.59 | 4 |  |  |  |
|  | Co | 0.11 |  | 3 | 0.77 | 0.02 | HF |
| *Apodemus flavicollis* | F |  | 0.67/0.59 | 5 |  |  |  |
|  | Co | 0.30 |  | 3 | 0.72 | 0.15 | S |
| *Myodes glareolus* (Slovakia) | F |  | 0.85/0.72 | 4 |  |  |  |
|  | Co | 0.29 |  | 3 | 0.99 | 0.99 | LS |
| *Micaelamys namaquensis* | F |  | 0.68/0.50 | 2 |  |  |  |
|  | Co | 0.24 |  | 2 | 0.75 | 0.98 | LS |
| *Rhabdomys pumilio* | F |  | 0.64/0.58 | 2 |  |  |  |
|  | Co | 0.04 |  | 2 | 0.99 | 0.99 | LS |
| *Grammomys ibeanus* | F |  | 0.79/0.67 | 4 |  |  |  |
|  | Co | 0.42 |  | 3 | 0.99 | 0.99 | LS |
| *Lophuromys kilonzoi* | F |  | 0.76/0.65 | 4 |  |  |  |
|  | Co | 0.46 |  | 4 | 0.42 | 0.02 | HF |
| *Mastomys natalensis* | F |  | 0.62/0.51 | 2 |  |  |  |
|  | Co | 0.01 |  | 2 | 0.66 | 0.99 | LS |
| *Praomys delectorum* | F |  | 0.62/0.51 | 4 |  |  |  |
|  | Co | 0.24 |  | 3 | 0.94 | 0.42 | S |

**Appendix 2. Scheme of constructing presence/absence matrices of flea-host interactions for different hierarchical scales**

1. Compound communities at the scale of a biogeographic realm

1a. Flea distribution among regions (within a biogeographic realm)

|  | Region 1 | Region 2 | Region 3 |
| --- | --- | --- | --- |
| Flea species 1 |  |  |  |
| Flea species 2 |  |  |  |
| Flea species 3 |  |  |  |

1b. Fleas distribution among host species (within a biogeographic realm)

|  | Host species 1 | Host species 2 | Host species 3 |
| --- | --- | --- | --- |
| Flea species 1 |  |  |  |
| Flea species 2 |  |  |  |
| Flea species 3 |  |  |  |

2. Compound communities at a regional scale

2a. Flea distribution among sampling sites (within a region)

|  | Site 1 | Site 2 | Site 3 |
| --- | --- | --- | --- |
| Flea species 1 |  |  |  |
| Flea species 2 |  |  |  |
| Flea species 3 |  |  |  |

2b. Fleas distribution among host species (within a region)

|  | Host species 1 | Host species 2 | Host species 3 |
| --- | --- | --- | --- |
| Flea species 1 |  |  |  |
| Flea species 2 |  |  |  |
| Flea species 3 |  |  |  |

3. Component communities

Distribution of flea species harboured by the same host species among sampling sites (within a region)

|  | Site 1 | Site 2 | Site 3 |
| --- | --- | --- | --- |
| Flea species 1 |  |  |  |
| Flea species 2 |  |  |  |
| Flea species 3 |  |  |  |

4. Infracommunities

Distribution of flea species harboured by the same host species at the same sampling site

|  | Host individual 1 | Host individual 2 | Host individual 3 |
| --- | --- | --- | --- |
| Flea species 1 |  |  |  |
| Flea species 2 |  |  |  |
| Flea species 3 |  |  |  |

**Appendix 3. Supplementary figures.**

**Fig. S1.** Modules based on trait similarity and spatial (across localities) co-occurrence similarity for component communities of fleas harboured by *Abrothrix olivacea* in Patagonia. The number inside or near the circle is the number of species in the module. In trait-associated modules, the number in parentheses is average within-module similarity (above line) and between-module similarity (below line) between pairs of species. Edge width is proportional to average similarity between species belonging to the modules.





**Fig. S2.** Modules based on trait similarity and similarity of co-occurrence across host individuals for infracommunities of fleas harboured by *Lophuromys kilonzoi* in Grewal (Gologolo, Tanzania). The number inside or near the circle is the number of species in the module. In trait-associated modules, the number in parentheses is average within-module similarity (above line) and between-module similarity (below line) between pairs of species. Edge width is proportional to average similarity between species belonging to the modules.
